# Effectiveness of deep versus moderate muscle relaxation during laparoscopic donor nephrectomy in enhancing postoperative recovery: study protocol for a randomized controlled study

**DOI:** 10.1186/s13063-017-1785-y

**Published:** 2017-03-04

**Authors:** Moira H. D. Bruintjes, Andries E. Braat, Albert Dahan, Gert-Jan Scheffer, Luuk B. Hilbrands, Frank C. H. d’Ancona, Rogier A. R. T. Donders, Cornelis J. H. M. van Laarhoven, Michiel C. Warlé

**Affiliations:** 1 Department of Surgery, Division of Vascular and Transplant Surgery, Radboud University Medical Center, Geert Grooteplein 10, 6525 GA Nijmegen, The Netherlands; 2 Department of Surgery, Leiden University Medical Center, Albinusdreef 2, 2333 ZA Leiden, The Netherlands; 3 Department of Anesthesiology, Leiden University Medical Center, Albinusdreef 2, 2333 ZA Leiden, The Netherlands; 4 Department of Anesthesiology, Radboud University Medical Center, Geert Grooteplein 10, 6525 GA Nijmegen, The Netherlands; 5 Department of Nephrology, Radboud University Medical Center, Geert Grooteplein 10, 6525 GA Nijmegen, The Netherlands; 6 Department of Urology, Radboud University Medical Center, Geert Grooteplein 10, 6525 GA Nijmegen, The Netherlands; 7 Department for Health Evidence, Radboud University Medical Center, Geert Grooteplein 10, 6525 GA Nijmegen, The Netherlands; 8 Department of Surgery, Division of General Surgery, Radboud University Medical Center, Geert Grooteplein 10, 6525 GA Nijmegen, The Netherlands

**Keywords:** Deep neuromuscular block, Early quality of recovery, Laparoscopic donor nephrectomy, Randomized controlled trial, Rocuronium

## Abstract

**Background:**

Postoperative recovery after live donor nephrectomy is largely determined by the consequences of postoperative pain and analgesia consumptions. The use of deep neuromuscular blockade has been shown to reduce postoperative pain scores after laparoscopic surgery. In this study, we will investigate whether deep neuromuscular blockade also improves the early quality of recovery after live donor nephrectomy.

**Methods:**

The RELAX-study is a phase IV, multicenter, double-blinded, randomized controlled trial, in which 96 patients, scheduled for living donor nephrectomy, will be randomized into two groups: one with deep and one with moderate neuromuscular blockade. Deep neuromuscular blockade is defined as a post-tetanic count of 1–2. Our primary outcome measurement will be the Quality of Recovery-40 questionnaire (overall score) at 24 h after extubation.

**Discussion:**

This study is, to our knowledge, the first randomized study to assess the effectiveness of deep neuromuscular blockade during laparoscopic donor nephrectomy in enhancing postoperative recovery. The study findings may also be applicable for other laparoscopic procedures.

**Trial registration:**

clinicaltrials.gov, NCT02838134. Registered on 29 June 2016.

**Electronic supplementary material:**

The online version of this article (doi:10.1186/s13063-017-1785-y) contains supplementary material, which is available to authorized users.

## Background

As patients with end-stage kidney disease and society both benefit tremendously from living kidney donors, their safety and wellbeing are highly important objectives in living kidney donation. A systematic review of the Cochrane collaboration by Wilson *et al.* has shown that laparoscopic donor nephrectomy is associated with reduced analgesia use, shorter hospital stay, and faster return to normal physical functioning, when compared with open donor nephrectomy [1]. Nowadays, the laparoscopic technique has become the gold standard in procuring live donor kidneys.

Advances in minimally invasive surgery for live kidney donors have led to the development of new techniques (i.e. laparoendoscopic single-site donor nephrectomy, or hand-assisted or robotic-assisted donor nephrectomies). Nevertheless, there is no evidence of superiority of these techniques as compared with standard laparoscopic donor nephrectomy [2–6].

Postoperative recovery is largely determined by the consequences of postoperative pain and analgesia consumption. In a previous pilot study by our group, low-pressure pneumoperitoneum (7 mmHg) during laparoscopic donor nephrectomy reduced postoperative pain scores, compared with standard pressure pneumoperitoneum [7]. This finding is in agreement with a meta-analysis showing that the use of low-pressure pneumoperitoneum provides a clinically relevant reduction in postoperative pain scores after laparoscopic surgery [8]. To investigate whether the use of low-pressure pneumoperitoneum facilitated by profound muscle relaxation improves not only postoperative pain but also the early quality of recovery, we performed a blinded randomized controlled trial in patients undergoing laparoscopic donor nephrectomy ([9], Ozdemir-van Brunschot DM, *et al.*: Low pressure pneumoperitoneum facilitated by deep neuromuscular blockade during laparoscopic donor nephrectomy is associated with reduced length of hospital stay, submitted). Results from this study showed that the use of low-pressure pneumoperitoneum did not reduce pain scores nor improve the quality of recovery (Ozdemir-van Brunschot DM, *et al.*: Low pressure pneumoperitoneum facilitated by deep neuromuscular blockade during laparoscopic donor nephrectomy is associated with reduced length of hospital stay, submitted). A possible explanation for this finding may be that the use of a deep neuromuscular blockade reduces pneumoperitoneum-related pain scores by increasing the compliance of the abdominal wall musculature. As deep neuromuscular blockade was applied in both arms of the study, this may have abated the analgesic effects of low-pressure pneumoperitoneum. Therefore, we hypothesize that the use of deep neuromuscular blockade during standard pressure laparoscopic donor nephrectomy improves the early quality of recovery after laparoscopic donor nephrectomy by reducing postoperative pain scores or analgesic consumption.

## Methods/Design

**Fig. 1:**
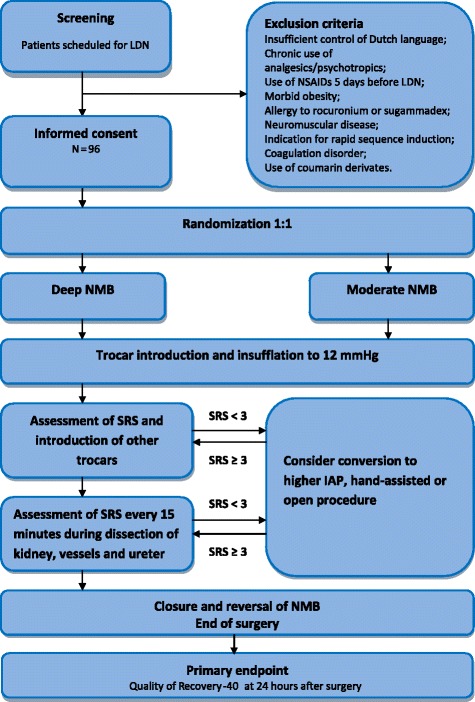
Flow chart. IAP, intra-abdominal pressure; LDN, live donor nephrectomy; NMB, neuromuscular blockade; NSAID, nonsteroidal anti-inflammatory drug; SRS, surgical rating scale

This multicenter, double-blinded, randomized controlled trial will be performed at the Radboud University Medical Center and the Leiden University Medical Center. We aim to assess the effectiveness of deep versus moderate neuromuscular blockade during laparoscopic donor nephrectomy in enhancing postoperative recovery. All eligible donors will be screened. Patients will be included after written informed consent. See Fig. 1 for the flow chart, Additional file 1 for the SPIRIT checklist, and Additional file 2 for the SPIRIT figure for study protocols.

### Study population

A total of 96 patients will be randomized, based on a computer-generated list, to either deep neuromuscular blockade (group A) or moderate neuromuscular blockade (group B). Stratification by center will be used. All adult individuals (>18 years), who are scheduled for living kidney donation are eligible for this study. Patients are excluded if they meet one or more of the following exclusion criteria: insufficient control of the Dutch language to read the patient information and to fill out the questionnaires; chronic use of analgesics or psychotropic drugs; use of nonsteroidal anti-inflammatory drugs <5 days before surgery; morbid obesity (body mass index > 35 kg/m^2^); known or suspect allergy to rocuronium or sugammadex; neuromuscular disease; indication of rapid sequence induction; deficiency of vitamin K-dependent clotting factors, coagulopathy or use of coumarin derivatives because of an increased risk of bleeding when combined with sugammadex; or peri-operative use of fusidic acid or flucloxacillin because of an interaction with sugammadex.

### Study protocol

Before the surgeons arrive at the operation room, all study medications are prepared by the anesthesiologist or the anesthesiologist’s assistant, after opening the envelope containing the allocation of treatment. Surgeons, scrub nurses, and the research physician are blinded for group allocation. Covering of the neuromuscular monitoring equipment, nerve stimulator and computer behind sterile drapes ensures this. The attending anesthetic staff in the operating room are not blinded as the anesthesiologist has to respond adequately to the neuromuscular monitoring to maintain adequate neuromuscular blockade according to the allocation of treatment.

Neuromuscular function will be monitored in a standardized fashion using an acceleromyograph at the wrist (train-of-four watch SX, MIPM GmbH). To calibrate the train-of-four watch, first a tetanic ulnar nerve stimulus (50 Hz for 5 s) will be administered. Thereafter, the train-of-four watch will be calibrated, followed by three measurements to ensure that the train-of-four ratio differs by less than 5%. If the train-of-four ratio differs by more than 5%, the train-of-four watch will be recalibrated. The post-tetanic count is obtained by counting 1 Hz twitches 3 s after 5 s of 50 Hz tetany. The post-tetanic count is used to assess the level of the deep neuromuscular blockade and to give an approximate time until the return of response to single twitches.

Anesthesia will be induced with remifentanil 1.0 μg/kg, propofol 1–2 mg/kg and rocuronium (intubation dose 0.6 mg/kg). Anesthesia is maintained with propofol aimed at a bispectral index score between 45 and 55; remifentanil administration will be at the discretion of the attending anesthesiologist. Tracheal intubation is performed 2 min after administration of 0.6 mg/kg rocuronium in both groups. For a body mass index > 30 kg/m^2^, the dose of rocuronium will be adjusted taking into account ideal body weight. We will use pressure-regulated volume-controlled ventilation through an endotracheal tube with a mixture of oxygen in air with 5 cm H_2_O positive end-expiratory pressure and tidal volume between 6 and 8 ml/kg. Minute ventilation is adjusted to main end-tidal carbon dioxide between 31 and 43 mmHg by changing respiratory rate during surgery. A nasogastric tube will be inserted for gastric decompression and will be removed before the end of the surgery. The core temperature will be measured continuously, with the aim at maintaining it at 36–37 °C.

In group A (deep neuromuscular blockade), infusion of rocuronium (0.3 to 0.4 mg/kg) is started and titrated towards post-tetanic count 1–2. In group B (moderate neuromuscular blockade), no additional rocuronium is administered after tracheal intubation and neuromuscular function will be allowed to recover spontaneously.

All laparoscopic procedures will be performed by two surgeons with at least one experienced transplant surgeon (>30 laparoscopic donor nephrectomies). The primary surgeon assesses Leiden surgical space conditions using the Leiden surgical rating scale according to Martini *et al.* [10] (see Table 1) after introduction of trocars, and then every 15 min. If surgical conditions are insufficient (surgical rating scale 1 or 2), the surgeon only decides to convert to a higher intra-abdominal pressure or an open- or hand-assisted procedure, if the safety of the patient is compromised. In case of insufficient surgical conditions due to (severe) muscle contractions (surgical rating scale 1 or 2), the protocol allows a 0.6 mg/kg bolus of rocuronium. Blood loss will be compensated with colloid solution; for 100 ml blood loss, 120 ml colloid solution is given. Surgical wounds will be locally infiltrated with 10 mL ropivacaine and the use of drains is avoided. After skin closure, the neuromuscular blockade is reversed with sugammadex, at a dose of 4 mg/kg in group A and a dose of 2 mg/kg in group B. When the patients have a stable train-of-four ratio of more than 0.9 for 2 min and are fully awake, extubation is performed. The blinded research physician will register intra-operative parameters (e.g. Leiden surgical rating scale score, blood loss, warm ischemia time, conversion to open or hand-assisted donor nephrectomy and intra-operative complications).

**Table 1:** Assessment of Leiden surgical space conditions (Leiden surgical rating scale) [10]

Scale		Description
1	Extremely poor conditions	The surgeon is unable to work because of coughing or the inability to obtain a visible laparoscopic field because of inadequate muscle relaxation.
2	Poor conditions	There is a visible laparoscopic field, but the surgeon is severely hampered by inadequate muscle relaxation with continuous muscle contractions, movements or both with the hazard of tissue damage.
3	Acceptable conditions	There is a wide visible laparoscopic field but muscle contractions, movements or both occur regularly, causing some interference with the surgeon’s work.
4	Good conditions	There is a wide laparoscopic field with sporadic muscle contractions or movements, or both.
5	Optimal conditions	There is a wide visible laparoscopic working field without movement or contractions.

Postoperative pain and nausea will be assessed at 1, 6, 24, and 48 h after extubation. Postoperative pain management is achieved by acetaminophen (total 4000 mg daily) and either patient-controlled analgesia (morphine, 1 mg morphine per bolus, lock-out 6 min) or intramuscular morphine, at the patient’s request. On day 1, patient-controlled analgesia will be replaced with oral analgesics. If there is postoperative nausea or vomiting, 4 mg intravenous ondansetron (maximum 12 mg/day) will be administered or, as an alternative, 10 mg intravenous metoclopramide (maximum 30 mg/day). On day 1, the urine catheter will be removed and a normal diet and mobilization will be started immediately. The research physician will assess all postoperative pain scores and perform daily evaluation with regard to the use of analgesics and anti-emetics and urine output. Discharge criteria will be evaluated daily. Discharge criteria are adequate pain control with oral medication, passage of flatus or defecation, intake of solid food tolerated, patient is mobilized and independent and patient accepts discharge. If hospitalization is prolonged for non-medical reasons (e.g. social reasons) the ‘virtual’ discharge date is listed.

### Outcome measures

The primary outcome measure is the total score of the Quality of Recovery-40 Questionnaire (QoR-40) at 24 h after extubation. This questionnaire has 40 questions measuring five dimensions: patient support, comfort, emotions, physical independence, and pain. Each item is rated on a scale of 1 to 5, giving a minimum score of 40 and a maximum score of 200 [11]. The QoR-40 is one of the most thoroughly validated assessment tools available for measuring a patient’s self-assessed quality of recovery after surgery [12].

The secondary outcome measures are: intra-operative parameters (e.g. surgical conditions (see Table 1), operation time, length of pneumoperitoneum, warm ischemia time, estimated blood loss, conversion to higher intra-abdominal pressure, hand-assisted or open donor nephrectomy, intra-operative complications), the total QoR-40 score at 48 h after extubation, postoperative pain (components of pain scores); postoperative nausea and vomiting (numeric rating scale), cumulative use of analgesics and anti-emetics, time to reach discharge criteria, re-admissions and postoperative complications. Follow-up will be performed after 30 and 60 days, evaluating the pain scores, complications and administering the Work and Healthcare Questionnaire.

The extent of pain, nausea and vomiting and cumulative analgesia and anti-emetics use will be assessed at 1, 6, 24, and 48 h. Complications and discharge criteria will be evaluated daily. A complete overview of the time schedule is given in Table 2.

**Table 2:** Overview of variables and time-points

	−18 h	1 h	6 h	24 h	48 h	30 days
Quality of Recovery-40 questionnaire	x			x	x	
Components of pain	x	x	x	x	x	x
Analgesia use	x	x	x	x	x	
Discharge criteria			x	x	x	
Evaluation of complications				x	x	x

### Adverse events and reactions

Since the use of a deep neuromuscular blockade with a standard insufflations pressure improves the quality of the surgical field, there are no risks related to the surgery [10]. Preliminary data from our recently performed Leopard-3 study show that the mean surgical rating scale was significantly better during deep neuromuscular blockade with less opiate consumption (Ozdemir-van Brunschot DM, *et al.*: Deep neuromuscular blockade improves surgical conditions during low-pressure pneumoperitoneum laparoscopic donor nephrectomy, submitted). A deep neuromuscular block can be achieved by higher doses of rocuronium as compared with the routine intubation dose. To overcome the extended effects of deep neuromuscular blockade that might lead to airway obstruction, hypoxia, pneumonia, or atelectasis, sugammadex is administered to antagonize the effects of rocuronium. Sugammadex can be administered safely to patients, without dose adjustments for young, elderly, or obese patients and can also be safely administered to patients with suboptimal renal function. Patients will only be extubated when the train-of-four is >90%. Furthermore, patients will remain in the post-anesthesia care unit for 2 h, to ensure adequate neuromuscular function.

### Sample size calculation

A sample size of 48 patients per group is needed to provide 90% power to detect a 10-point difference in the quality of recovery score at 1 day after extubation (alpha, 5%). Based on our previous studies, the standard deviation is 15 points [9].

It is generally assumed that a clinically relevant improvement in quality of recovery is represented by a point 10 difference in QoR-40 score, because a 10-point difference represents a 15% improvement in the quality of recovery based on previously reported values on the mean and range of the QoR-40 score in patients after anesthesia and surgery [12–17].

### Data management and monitoring

Subjects will be coded by a numeric code to create an anonymous dataset. A Castor database will be developed and used for data management. Monitoring will be conducted in accordance with negligible risk monitoring guidelines of the Dutch Federation of Academic Medical Centers. The investigator will submit a summary of the progress of the trial to the accredited medical research ethics committee once a year. Information will be provided on the date of inclusion of the first subject, numbers of subjects included and numbers of subjects who have completed the trial, serious adverse events, serious adverse reactions, other problems and amendments.

### Statistical methods

For the primary analysis, groups A and B will be compared with regard to the primary end-point (QoR-40 score at day 1). Analysis of covariance (ANCOVA) will be used to compare groups and to adjust for co-variates i.e. age, sex, number of arteries, and side of nephrectomy. Statistical significance will be considered for *P* < 0.05. All analyses will be performed on an intention-to-treat basis. When a significant number of patients (>10%) allocated to a moderate neuromuscular blockade have received deep relaxation due to additional boluses of rocuronium (post-tetanic count ≤ 2) for the duration of >50% of the operation time, both an intention-to-treat and a per-protocol analysis will be performed. Statistical analyses will be performed using SPSS 22.0 (SPSS Inc., Chicago, IL, USA).

## Discussion

Laparoscopic donor nephrectomy has become the gold standard in procuring live donor kidneys. Laparoscopic donor nephrectomy is associated with reduced analgesia use, shorter hospital stay, and faster return to normal physical functioning, as compared with open donor nephrectomy. Originally, enhanced recovery programs have been developed in Europe to address prolonged length of stay after colorectal resections [18]. The enhanced recovery program care pathways reduce surgical stress, maintain postoperative physiological function, and enhance mobilization after surgery. It has been shown that enhanced recovery programs within laparoscopic surgeries decrease the length of stay, with equivalent morbidity, mortality, and readmission rates [19]. Likewise, Waits *et al.* showed that implementation of an enhanced recovery program in living donor nephrectomy improves length of stay and narcotic use, as compared with a standard care protocol [20]. In our previous trials, we studied the effect of low-pressure pneumoperitoneum during laparoscopic donor nephrectomy on postoperative pain scores and the early quality of recovery. Live kidney donors allocated to the low-pressure group showed lower postoperative pain scores.

The use of a deep neuromuscular block could be a new element of an enhanced recovery program in patients undergoing laparoscopic surgery. Our primary hypothesis is that the use of deep neuromuscular blockade during laparoscopic donor nephrectomy improves the early quality of recovery of living donor kidney donors. Deep neuromuscular blockade may increase relaxation of the abdominal wall musculature; this may reduce stretch-related abdominal pain scores after laparoscopy. Some evidence exists that the use of a deep neuromuscular blockade reduces pain scores independently of the insufflation pressure. For example, Martini *et al.* showed that deep neuromuscular blockade improves surgical conditions, with reduced postoperative pain scores after laparoscopic surgery [10]. Moreover, preliminary data of our recently performed study (Ozdemir-van Brunschot DM, *et al.*: Deep neuromuscular blockade improves surgical conditions during low-pressure pneumoperitoneum laparoscopic donor nephrectomy, submitted) show that the mean surgical rating scale was significantly better during deep neuromuscular blockade, with less opiate consumption.

The main strength of our study is that this is the first randomized controlled trial designed to study the effect of deep neuromuscular blockade on the early quality of recovery. The QoR questionnaire is a thoroughly validated assessment tool to measure a patient’s self-assessed quality of recovery after surgery [12]. Improvement in the early quality of recovery increases patients’ comfort and may contribute to earlier eating and mobilization. According to the principles of enhanced recovery, this may lead to a reduction in hospital stay. Another strength is that we study a highly homogeneous population of relatively healthy kidney donors. This may reduce bias, as similar baseline characteristics in both groups decrease the risk of confounding factors. Finally, it is reasonable to assume that results from this study also hold for many other laparoscopic procedures.

A limitation of this study is that the minimal clinically important difference of the quality of recovery score is still under debate. We performed a power calculation based on the assumption that the minimal clinically important difference is 10 points. Recently Myles *et al.* published data [21], in which they estimate a 6.3 point difference as the minimal clinically important difference for the QoR-40 score. This estimate was based on triangulation of distribution- and anchor based calculations of the minimal clinically important difference. However, it is important to note that the study population included a broad range of surgical procedures, varying in extent as well as type of surgery. In our view, a 10-point difference in the QoR-40 score would be more appropriate than a 6.3 difference, as the gain in quality of recovery has to outweigh the additional costs and expertise required for the application of deep neuromuscular blockade.

In conclusion, we aim to investigate the effects of deep neuromuscular blockade on the early quality of recovery in laparoscopic donor nephrectomy. Our hypothesis is that the use of deep neuromuscular blockade will lead to enhanced recovery after surgery.

### Trial status

Recruiting patients.

## Additional files

Additional file 1: SPIRIT checklist. (DOC 123 kb)
Additional file 2: SPIRIT figure. (DOC 55 kb)

## Abbreviations

ANCOVA: Analysis of covariance
QoR-40 questionnaire: Quality of Recovery-40 questionnaire
SPIRIT: Standard Protocol Items: Recommendations for Interventional Trials
