# Supplementary material for: Effectiveness of deep versus moderate muscle relaxation during laparoscopic donor nephrectomy in enhancing postoperative recovery: study protocol for a randomized controlled study
Source: Trials. 2017 Mar 4;18:99. doi: 10.1186/s13063-017-1785-y (PMC5336688; doi:10.1186/s13063-017-1785-y)
Supplement: Additional file 2: — SPIRIT figure. (DOC 55 kb) [file 13063_2017_1785_MOESM2_ESM.doc]

SPIRIT figure. Schematic overview of enrolment, interventions, and assessments

|  | **STUDY PERIOD** | | | | | | | | |
| --- | --- | --- | --- | --- | --- | --- | --- | --- | --- |
|  | **Enrolment** | **Allocation** | **Post-allocation** | | | | | | **Close-out** |
| **TIMEPOINT** | ***1 day before surgery*** | **Day of surgery**  **(at OR)** | ***+1 h*** | ***+6h*** | ***+24h*** | ***+48h*** | ***+72h*** | ***+4***  ***weeks*** | ***+8 weeks*** |
| **ENROLMENT:** |  |  |  |  |  |  |  |  |  |
| **Eligibility screen** | X |  |  |  |  |  |  |  |  |
| **Informed consent** | X |  |  |  |  |  |  |  |  |
| **Allocation** |  | X |  |  |  |  |  |  |  |
| **INTERVENTIONS:** |  | During surgery |  |  |  |  |  |  |  |
| ***Deep NMB*** |  |  |  |  |  |  |  |  |  |
| ***Moderate NMB*** |  |  |  |  |  |  |  |  |  |
| **ASSESSMENTS:** |  |  |  |  |  |  |  |  |  |
| ***Baselines characteristics*** | X |  |  |  |  |  |  |  |  |
| ***L-SRS*** |  |  |  |  |  |  |  |  |  |
| ***QOR-40*** | X |  |  |  |  |  |  |  |  |
| ***Pain scores*** | X |  | X | X | X | X | X | X | X |
| ***Analgesia use*** | X |  | X | X | X | X | X |  |  |
| ***Discharge criteria*** |  |  |  | X | X | X | X |  |  |
| ***Complications*** |  | X | X | X | X | X | X | X | X |

NMB = neuromuscular blockade; L-SRS = Leiden-Surgical Rating Scale; QOR-40 = Quality of Recovery-40 questionnaire.
